# Blockchained supply chain management based on IoT tracking and machine learning

**DOI:** 10.1186/s13638-022-02209-0

**Published:** 2022-12-29

**Authors:** Zhongping Dong, Wei Liang, Yan Liang, Weibo Gao, Yi Lu

**Affiliations:** China Telecom Research Institute, Beijing, China

**Keywords:** Internet of things (IoT), Supply chains, Blockchain, Machine learning (ML), Big data, GRU, Multi-head attention

## Abstract

When it comes to running and managing modern supply chains, 6G Internet of things (IoT) is of utmost importance. To provide IoT with security and automation, blockchain and machine learning are two upper-layer technology that can help. First, we propose to utilize blockchain in modern supply chains to ensure efficient collaboration between all parties. Second, we adopt multi-head attention (MHA)-based gated recurrent unit (GRU) to do inbound logistics task prediction. Finally, numerical results justify that multi-head attention-based GRU model has better fitting efficiency and prediction accuracy than its counterparts.

## Introduction

The manufacturing sector has been profoundly affected by globalization, increasing product life-cycle dynamics, and mass customization [1, 2]. To cut down on production and administration costs, most contemporary manufacturers now contract out to third-party vendors for the creation of individual components. The primary focuses of a contemporary manufacturer are product development, integration, and marketing. Critical steps in the supply chain process include sourcing and manufacturing components, assembling finished goods, and moving them to retail outlets for sale [3]. The supply chain also in-corporates supplier management and product management.

The key to improving the competitiveness of modern manufacturing companies lies in improving the efficiency of their production and operations, especially effective product quality control. Production plan changes, poor logistics and rework caused by product defects can seriously affect supply chain management. Therefore, modern enterprise supply chain management should actively shift from reactive response to proactive prevention. However, proactive prevention of supply chain management may result in higher management costs as well as redundancy and inefficiency of the system [4]. Currently, product quality inspection is generally analyzed subjectively by professional technicians using qualitative methods. However, using predictive models to predict the quality of products in advance and to ensure continuous improvement of product quality may yield higher benefits in future.

Internet of things (IoT) has important implications for the development of supply management. IoT tracking technology has come a long way in the last decade. IoT tracking system contains four main components, IoT device tag, location system, location engine and wireless communication with the cloud platform. As location systems and wireless communication technologies have matured, IoT trackers can now be used to monitor the location of IoT products in real time. However, modern supply chain technology advances have driven the emergence of more complex and diverse industrial application scenarios with higher performance requirements, and IoT tracking solutions to meet the needs of these scenarios are still in their infancy. In the literature [5], the authors investigate a solution to the bullwhip effect based on IoT technology to ensure real-time transmission and sharing of information among parties in the supply chain. In the literature [6], the authors studied the application of IoT technology in logistics enterprises and analyzed the impact of IoT on various aspects of logistics management. In the literature [7], the authors comprehensively investigated the security issues and challenges in supply chain management. In the literature [8], the authors apply machine learning to supply management, which can maximize the value of information sharing and data flow by integrating ML technology into various tools for supply chain management.

Strong assistance from current network technology is essential for identifying, tracing, real-time tracking, and exchanging information about commodities in the supply chain. All things inside a territory may now be linked together and communicate with one another thanks to the Internet of Things and the 4G/5G public land mobile network. However, the terrestrial-network's scope of services is so narrow that it is unable to cover areas like deep space, deep sea, and the polar regions. A space-air-ground-sea network is a large-dimensional network with four tiers: a space-network tier made up of satellites, an air-network tier made up of flying BSs (e.g., high-altitude platforms, mobile airborne cells, UAVs, and so on), a terrestrial-network tier made up of legacy BSs, and an underwater-network tier made up of underwater hubs, ships, and so on.

The supply chain industry has undergone significant transformation throughout the years. Many players, who have historically been sluggish to adopt technology, are now experiencing the benefits of modern digital solutions, such as cloud computing and mobility. Identification, traceability, and real-time tracking of commodities in the supply chain have long been hampered by the heterogeneity of platforms and technologies employed. From product tracking to inventory management, the supply chain is prepared for its own IoT-driven upgrade.

The Internet of things enables the collection, transport, storage, and sharing of logistics information for improved supply chain partner cooperation and interoperability. Given the worry over the sustainability of quality in certain industries, such as the pharmaceutical supply chain, there is a great deal of focus on the regular monitoring and verification of quality assurance and quality of experience activities throughout the supply chain network. For IoT solutions to be successful in practice, they must meet supply chain needs and meet certain quality standards. Better-designed tracking and traceability systems and information models make it simple for parties to utilize the technology and positively impact the supply chain.

In the information society, almost every enterprise cannot do without supply chain network, and tracking and monitoring all aspects of product design, production and distribution, and sales through supply chain network is the fundamental guarantee for efficient operation of the enterprise. However, the management of supply chain network should not only complete the normal various functions, but also be able to deal with various abnormal situations and improve the security of the network in order to avoid enterprises from falling into some unpredictable risks. For example, due to the lack of supply chain security management, some criminals may take advantage of this loophole to engage in the production and sale of counterfeit or pirated goods. This will not only cause economic loss to consumers, but also affect the social reputation of the enterprise, and in serious cases, it may bring troublesome legal problems to the enterprise.

Blockchain has seen some early success in recent years in areas like trade finance and industrial Internet [9]. The security, privacy, and trust challenges relating to smart grids have recently received a lot of scholarly attention [10–19]. For instance, [10, 11] detailed the use of blockchain in the energy Internet and identified a number of issues that were caused by it. A review of blockchain applications for smart grids and new frameworks was presented in [12]. Typical blockchain use cases for energy applications, including distributed energy transactions, smart microgrids, smart power distribution, and smart power consumption, are discussed in [13]. Blockchain provides a decentralized trust framework for distributed energy operations. Further research was done on the peer-to-peer energy transaction using blockchain and it was utilized for dispatching distributed energy on the basis of [14–18]. Ahl et al. [19] provides an review of blockchain-based distributed energy.

In all aspects of modern enterprise supply chain management, a large number of networked devices, such as RFID readers, mobile communication devices, network cameras, and IoT terminals will generate massive amounts of data. Based on the analysis of these big data, companies can enhance the decision-making process in the supply chain and help companies make the best operating model and operational decisions. Because of this, blockchain will have a record amount of data, which will significantly accelerate the globalization of technologies. Blockchain will be possible to offer trustworthy tracking, tracing, and spread-out point-to-point transactional capabilities for billions of products throughout the world. The inadequacies of IoT's weak safety and privacy, lack of confidence in virtual exchanges, and insufficient protection of ownership rights can only be partially made up for by blockchain technology. The growth of IoT and new business models will be significantly aided by the decentralization of blockchain, preservation of the privacy of transaction information, prevention of tampering with historical data, and traceability.

Our goal is to forecast the future flow of incoming logistics using big data, machine learning, and blockchain technology for incoming logistics planning. The application of machine learning to a supply chain network can automate and simplify its administration by streamlining its operations. Specifically, machine learning can be used to estimate product demand and swiftly change logistics management to provide rapid responses to client requests. This research integrates machine learning into the logistics planning process based on business knowledge of inbound logistics planning [20, 21].

The reminder of this paper is organized as follows. Section II introduces the basics of supply chain management as well as the involvement of 5G IoT tracking, blockchain and machine learning. Section III develops an automatic approach to predict inbound logistics tasks by using Multi-Head Attention-Based GRU (MHA-GRU). Section IV presents the numerical results to justify the performance of our design, followed by Section V to conclude this paper.

## Blockchained supply chain management and machine learning

### 5G IoT-based supply chain management

**Fig. 1:**
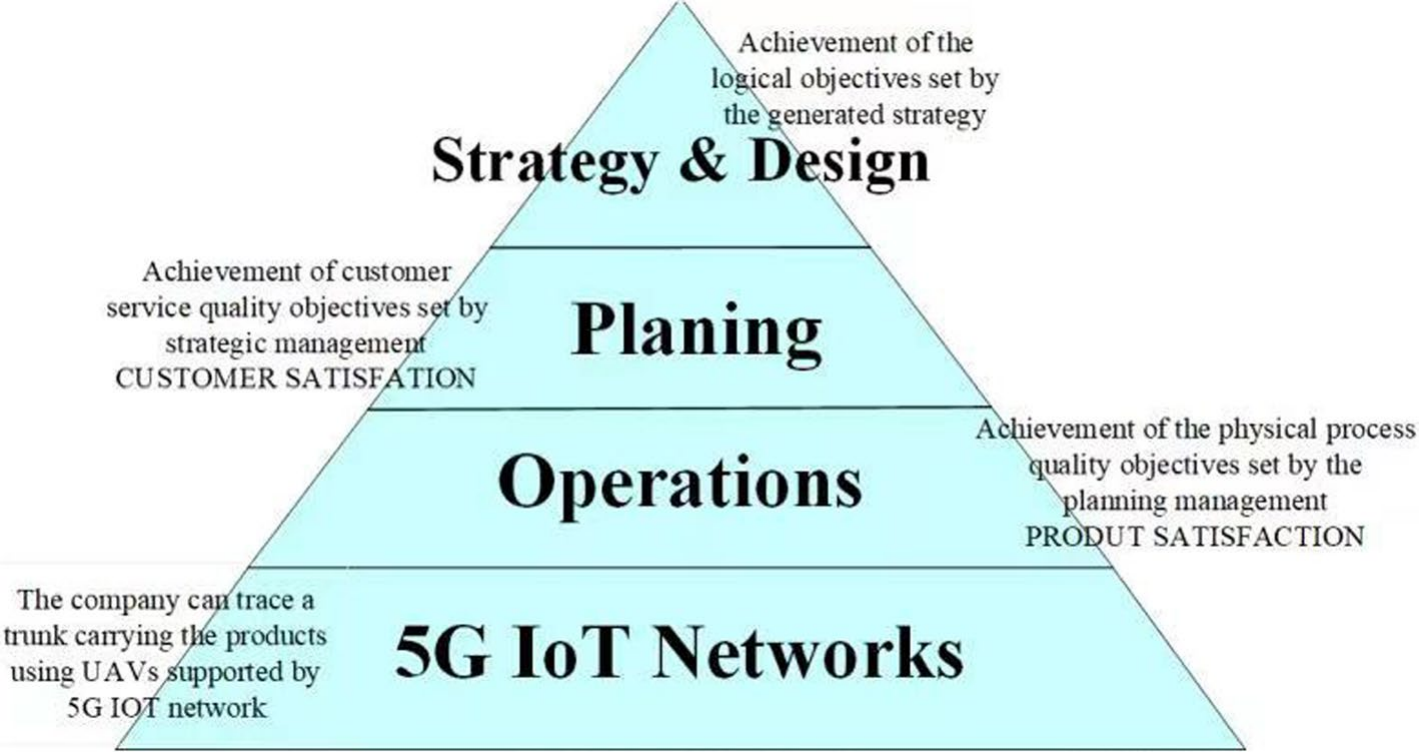
Four layers in supply chain management

In general, supply chain management includes three stages: supply chain design, supply chain planning, and supply chain operation, as shown in Fig. 1. Effective supply chain management often needs to take into account the uncertainty of decisions related to product and capital flows. In this respect, it would be significantly beneficial to bring 5G IoT as the infrastructure of the management.

- Supply Chain Design: It is a long-term decision made by the company according to its long-term development goals, and generally will not be changed in a short period of time, unless there is a major mistake in the management decision initially made. Such decisions are usually at the strategic level of the company's development, including both the company's internal operation strategy and the cooperation strategy with outsourcing companies, and are the backbone of the company's supply chain network.
- Supply Chain Planning: At this stage, the company integrates all short-term tasks under the designed supply chain framework and makes reasonable planning. These tasks include supply demand, inventory strategy, marketing target and price strategy. The purpose of this stage is to provide reasonable planning for short-term operational realization to ensure supply chain surplus. Moreover, if the planning stage can proceed smoothly, it shows that the strategic decision of supply chain design can be guaranteed.
- Supply Chain Operation: It is the stage of real-time operation of the supply chain network, which reflects the real-time flow of products at each node in the network. Depending on the speed of response and progress, the next node typically needs to make a corresponding decision quickly within minutes, hours, or days. For example, in the process from when a customer initiates an order request to when a product is received, multiple different network nodes may be designed, and each node responds according to its own status and received requests, and makes the next decision.
- 5G IoT Networks: The rapidly growing paradigm of Internet of Things will play a critical role in supply chain management. With 5G, airborne anchor nodes (AN) can provide relay services and aid in the localization of terrestrial IoT sensors. Using a group of UAVs outfitted with received signal strength indicator (RSSI) sensors, for instance, the enterprise may track a trunk carrying sensor-enabled merchandise, which can be considered a mobile IoT device with an unknown position.

### Challenges for supply chain

Modern enterprise supply chain management has become an important goal for the development of various industries, and researchers have applied big data, machine learning, IoT and blockchain technologies to supply chain management for better efficiency, reliability and security. However, modern enterprise supply chain management still faces the following challenges [7].

- The area covered by supply chain management will be unprecedentedly broad, covering not only emerging industries such as urban logistics and e-commerce, but also many traditional manufacturing and sales industries will establish modern supply chain networks. This requires the study of a common modern supply chain network model, and requires the model to have flexible industry applicability and high usability.
- Supply chain security management is an important goal of modern enterprise development. Unlike the traditional industry operation model, the supply chain management of modern information network-based enterprises faces many potential threats and security attacks. Therefore, it is necessary to establish a secure reference model for modern enterprise supply chain management so that enterprise data and transactions can be transmitted, exchanged and processed for analysis in a secure environment.
- Big data analytics and machine learning algorithms will play an increasingly important role in supply chain network management. Given the special needs of supply chain networks, the functional and performance specifications for large-scale data analytics and computer learning algorithms are more stringent. For example, there is an extremely high demand for privacy protection of data and security of transactions in supply chain networks, so big data analytics and machine learning algorithms need to have extremely high privacy protection capability and distributed security processing capability.

### Advantage of blockchain

The aforementioned drawbacks of insufficient property rights protection, insufficient security and privacy, and lack of confidence in virtual exchanges may only be partially remedied by blockchain technology. The growth of the IoT supply chain will be successfully aided by the decentralization of blockchain, transactional information, privacy security, and historical data anti-tampering in the smart grid.

Blockchain can greatly cut labor costs and transaction times by automating a variety of transaction operations. For instance, IBM employs blockchain technology to address the issues with contracts for temporary labor and has created equivalent solutions for invoice reconciliation to address the issues with invoices brought on by temporary labor. In addition to ensuring that payment conditions are followed and eliminating invoice dispute situations, reconciliation using a digital ledger may significantly lower reconciliation costs and accelerate work processes. Blockchain technology has given the corporate world a breath of new air by serving as a shared digital ledger for documenting transactions.

**Fig. 2:**
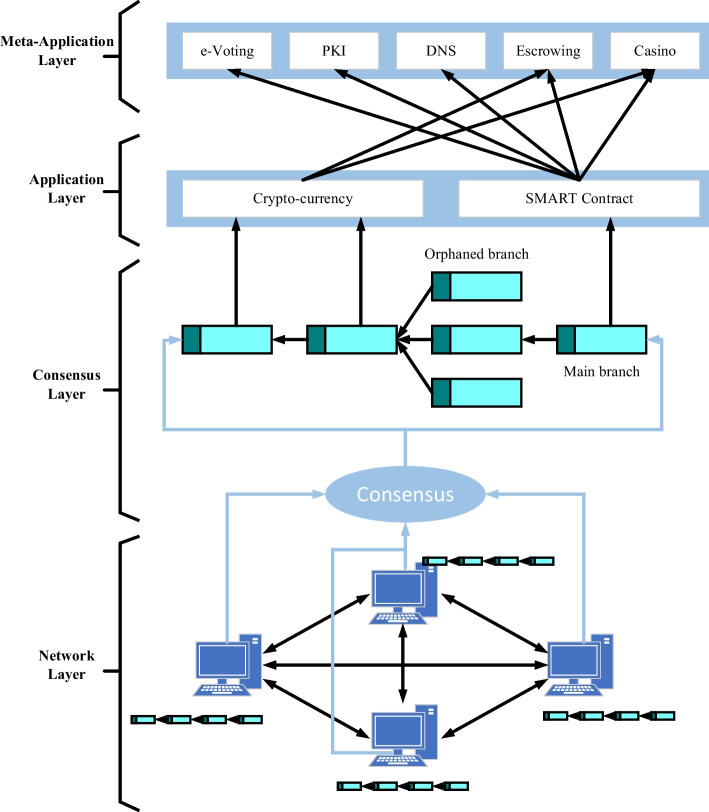
Layered structure of blockchain systems

From a layered perspective, the blockchain system consists of the network, consensus, application and meta-application layers, as shown in Fig. 2 [22]. The network layer is used to implement the blockchain network and information transfer among all nodes. The consensus layer allows highly decentralized nodes to agree on the validity of data in the blockchain network. The application layer encapsulates the various applications and scenarios of the blockchain, while the meta-application layer provides various standard basic function calls for the application layer.

### Machine learning applications in supply chain

The application of computer learning to supply chain management will be a major feature of modern supply chain network and an important guarantee to realize efficient operation of modern supply chain network. Traditional supply chain management has poor information integration and predictive analysis capabilities, while modern supply chain management based on machine learning and analysis of massive data will completely change this situation. Machine learning creates management models based on enterprise operations and business characteristics, and makes accurate production and scheduling decisions by analyzing the input big data. Further, the management model can also be readjusted to make the supply chain management better day by day by measuring the production and operation of the enterprise and the revenue status over a period of time. At the same time, local data offloading and access control issues for different system components will be resolved with the use of blockchain innovation to the supply chain [23]. When computer resources are few, it is possible to implement the dispersed deployment of a variety of resources to guarantee info on transaction traceability. The broad deployment of the mobile edge computing (MEC) servers will make it simple to manage IoT devices on the blockchain and save their information and data.

Machine learning is most commonly used in supply chain management for product demand forecasting and product production forecasting. Product demand information is derived from marketing, financial and production data, as well as other non-product factors, such as seasonality or “special events”. Based on this data and influencing factors, learning is used to accurately forecast customer demand for products. Further, the company adjusts the production status of the product based on the forecasted demand and the current product inventory and production capacity. Product production forecasting is used to measure the production capacity of a certain product and the ability of coordination and cooperation between different outsourcers. This is a very important management process in the supply chain. Accurate production forecasting helps companies to order raw materials and coordinate production plans for different products in a prompt and precise way, so that they can fulfill customer orders quickly and speed up product distribution and reduce inventory, thus reducing costs and increasing revenues.

Machine learning algorithms should be able to meet the dynamic and diverse demand applications. Different supply chain networks have different demand for applications and different time accuracy and location of demand changes. Therefore, the factors affecting demand forecasting and production forecasting are diverse, dispersed, complex and unstable, which poses a very serious challenge to machine learning-based supply chain management. In addition, due to some uncertain safety factors, machine learning needs to be safe training. Therefore, machine learning-based blockchain is an effective way to ensure the safety of machine learning model training process.

**Fig. 3:**
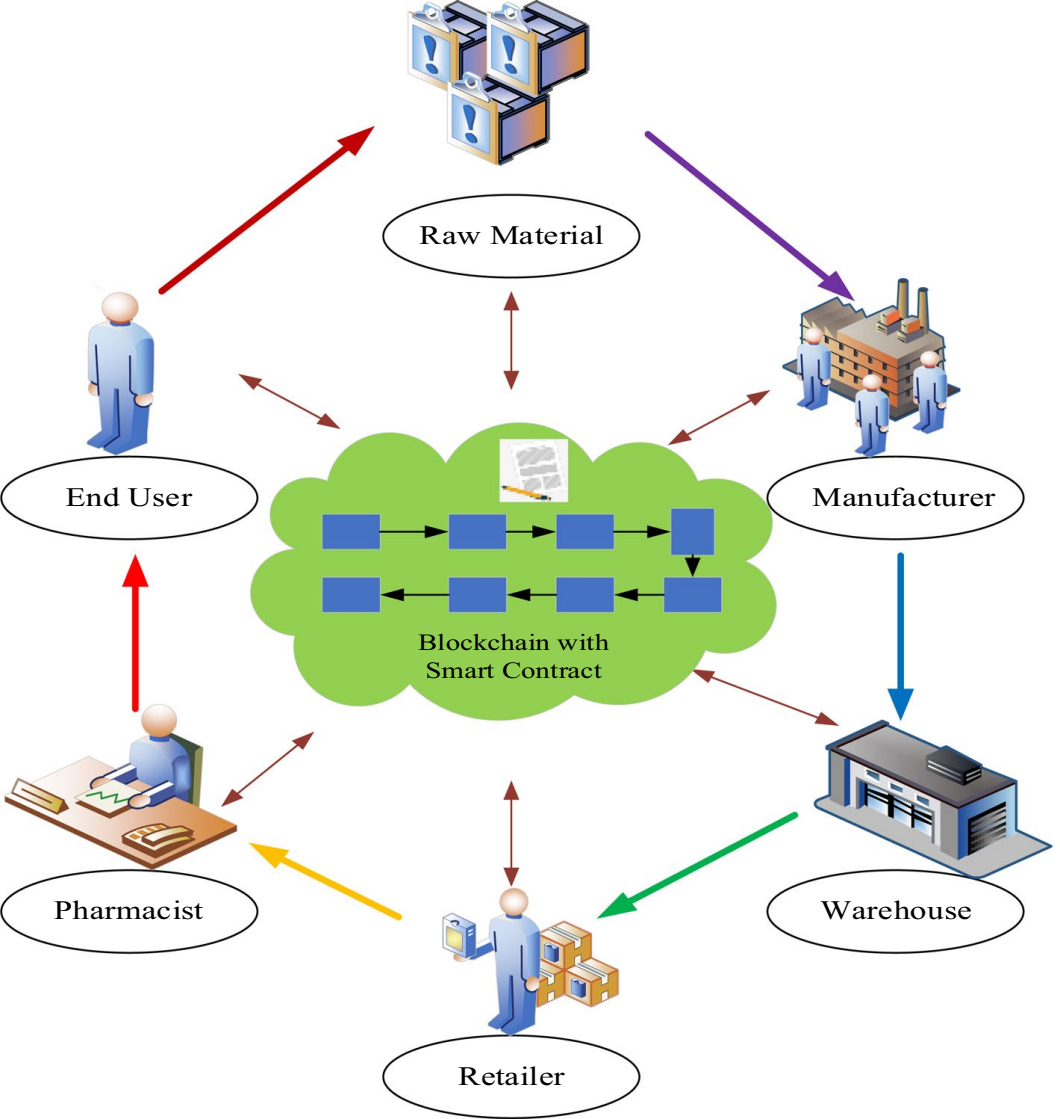
System architecture

Currently, in the context of the raging global COVID-19 epidemic, vaccinating the global population with COVID-19 vaccine is one of the challenging tasks faced in supply chain management. COVID-19 vaccine supply chain management should be manageable, operational, and auditable for both relevant government departments and stakeholders such as manufacturers, but also visible and accessible to the general public to gain their trust and support. The current vaccine supply chain fails to include vaccine manufacturers, distributors, hospitals, and government departments in this unified platform. This calls for a COVID-19 vaccine supply chain network based on big data analytics and machine learning to be launched as soon as possible to achieve success in the global immunization campaign (Fig. 3).

In COVID-19 vaccine supply chain management, machine learning will play an important role in predicting vaccination demand and managing vaccination regions and populations. For example, if big data analysis shows that the population in a certain region is more inclined to receive a specific vaccine, the supply chain network will have to make timely decisions based on this analysis to increase the production and targeted deployment of that vaccine. For example, if a certain vaccine has special requirements for transportation and storage conditions, the supply chain network should market and promote this vaccine to regions that meet the storage conditions and select logistics companies that meet the transportation conditions as partners.

Therefore, machine learning-based algorithms in the COVID-19 vaccine supply chain network need to consider a variety of factors, including country, region, vaccine manufacturer, vaccine storage requirements, number of vaccinated/unvaccinated people, and distribution of vaccinated regions. Different machine learning algorithms are suitable for accomplishing different specific functions. For example, using ML regression algorithms to accomplish vaccination demand prediction, while ML classification algorithms are better suited to accomplish vaccination selection. In addition, in order to transparently track COVID-19 vaccine distribution and protect the privacy and security of vaccinated population data, machine learning needs to be combined with other advanced technologies, such as building blockchain-based machine learning models. Decentralized machine learning built regards to blockchain technology will have additional benefits in terms of data security, identity verification, protection of privacy, and other areas, which will encourage and support the widespread deployment of machine learning application scenarios. As a matter of fact, the MEC server may be used to install the blockchain platform or application, enabling support for a variety of application scenarios.

## Methods

### GRU basics

Gate Recurrent Unit (GRU) is a kind of recurrent neural network. The GRU neural network is a variant of the LSTM neural network (long short-term memory network), which is based on the RNN (recurrent neural network), a well-established machine learning method that has great advantages in processing temporal series [24]. The RNN contains a signal feedback structure that correlates the output information at time *t* with the information before time *t*, and has dynamic features and memory functions.

**Fig. 4:**
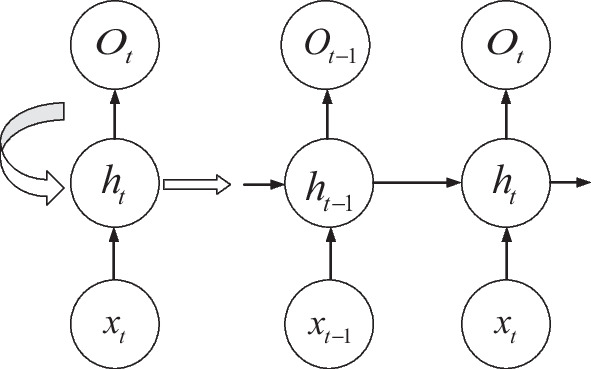
RNN structure diagram

**Fig. 5:**
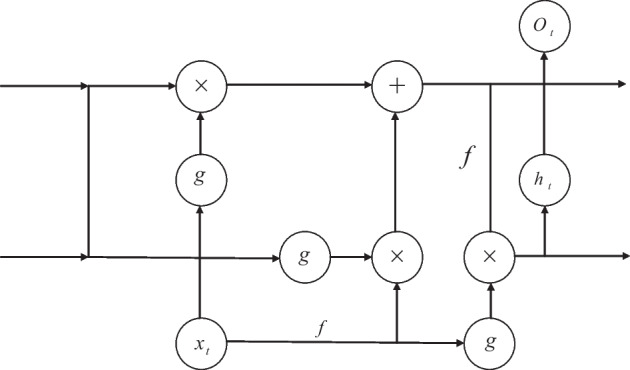
Structure diagram of LSTM hidden layer

Figure 4 shows the structure of the RNN. As can be seen from the figure: ① the RNN structure includes an input hidden layer and an output layer, where the hidden layer contains the feedback structure; ② the output value at time *t* is the result of the joint action of the input information at that time and the time before; ③ the RNN can effectively analyze and process short time series, but cannot analyze and process time series with too long a dimension, otherwise it will produce "gradient disappearance RNNs can effectively analyze and process shorter time-series, but cannot analyze and process time-series with too long a dimension, otherwise it will result in gradient disappearance or gradient explosion [25]. To solve this issue, [26] proposed an RNN improved structure LSTM neural network, whose hidden layer structure is shown in Fig. 5.The LSTM neural network achieves memory controllability in temporal order based on the memory units (forgetting gate, input gate and output gate) in the hidden layer, it resolves the issue of RNN's inadequate long-term memory, but its hidden layer structure is too complex and the sample training takes a lot of time [27]. Based on the LSTM neural network, [28] proposed a GRU neural network, using reset gates and update gates instead of forgetting gates, input gates and output gates in the LSTM neural network. Although the hidden layer data flows in the LSTM and GRU neural networks are comparable, the GRU neural network lacks a dedicated storage unit, making sample training more effective.

$h_{{\text{t}}}$ is the value of the hidden layer at moment *t*, and $h_{{{\text{t}} - 1}}$ is the value of the hidden layer before moment *t*; $o_{{\text{t}}}$ is the value of the output layer at moment *t*, and $o_{{{\text{t}} - 1}}$ is the value of the output layer before moment *t*; W is the value of the hidden layer previously used as this input's weight matrix, and the input layer to the hidden layer's weight matrix is represented by U., V represents the weight matrix from the output layer to the hidden layer; each circle represents a neuron. For a regular RNN hidden layer, when given the input value $x_{{\text{t}}} \left( {t = 1,2, \ldots ,n} \right)$, the output values of the output layer, hidden layer at moment *t* can be calculated by following a series of equations:

1 $$f = \tanh \left( x \right) = \frac{{e^{x} - e^{ - x} }}{{e^{x} + e^{ - x} }}$$

2 $$h_{{\text{t}}} = f\left( {U \cdot x_{{\text{t}}} + W \cdot h_{{{\text{t}} - 1}} } \right)$$

3 $$g = {\text{sigmoid}}\left( x \right) = \frac{1}{{1 + e^{ - x} }}$$

4 $$o_{{\text{t}}} = g\left( {V \cdot h_{{\text{t}}} } \right)$$

For a regular LSTM hidden layer, when given the input value $x_{{\text{t}}} \left( {t = 1,2, \ldots ,n} \right)$, the output values of the output layer, hidden layer at moment *t* can be calculated by following a series of equations.

5 $$f_{{\text{t}}} = g\left( {W_{{\text{f}}} \cdot h_{{{\text{t}} - 1}} + U_{{\text{f}}} \cdot x_{{\text{t}}} } \right)$$

6 $$i_{{\text{t}}} = g\left( {W_{i} \cdot h_{{{\text{t}} - 1}} + U_{i} \cdot x_{{\text{t}}} } \right)$$

7 $$a_{{\text{t}}} = f\left( {W_{{\text{a}}} \cdot h_{{{\text{t}} - 1}} + U_{{\text{a}}} \cdot x_{{\text{t}}} } \right)$$

8 $$c_{{\text{t}}} = c_{{{\text{t}} - 1}} \odot f_{{\text{t}}} + i_{{\text{t}}} \odot a_{{\text{t}}}$$

9 $$o_{{\text{t}}} = g\left( {W_{{\text{O}}} \cdot h_{{{\text{t}} - 1}} + U_{{\text{O}}} \cdot x_{{\text{t}}} } \right)$$

10 $$h_{{\text{t}}} = o_{{\text{t}}} \odot f\left( {c_{{\text{t}}} } \right)$$

where $f_{{\text{t}}}$, $i_{{\text{t}}}$ and $c_{{\text{t}}}$ represent forget gate, input gate and cell update, respectively. $W_{{\text{f}}}$, $W_{i}$ and $W_{{\text{a}}}$ represent the relationship coefficient of each gate.

**Fig. 6:**
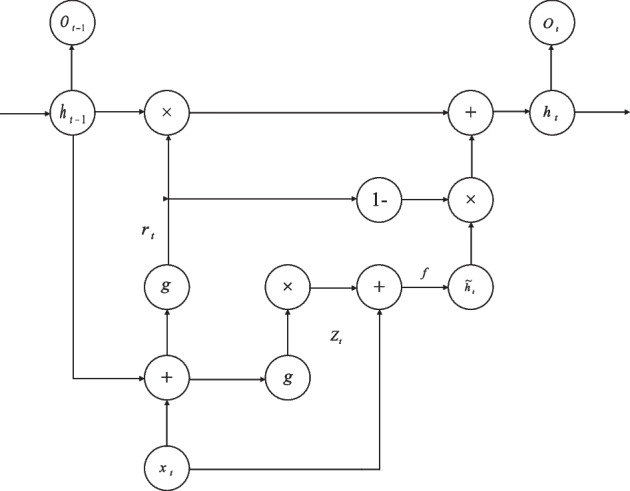
Structure diagram of GRU hidden layer

Figure 6 shows the hidden layer structure of GRU neural network. As can be seen from the diagram: the update gate regulates how much information from a prior instant influences the information in the present moment; the greater the update gate's value, the less influence past knowledge has on the present. The reset gate regulates how much information is taken in from the previous instant; the higher the value of the reset gate, the more information is taken in.

"1-" means that the vector's elements are each deducted by one. The hidden layer's value $h_{{\text{t}}}$ at time *t* is more likely to be impacted by the candidate value $\tilde{h}_{{\text{t}}}$ at time *t* than by the hidden layer's value $h_{{{\text{t}} - 1}}$ at time *t*-1 if the update gate's value $z_{{\text{t}}}$ at time *t* is bigger. If the value of $z_{{\text{t}}}$ is taken to be approximately 1, this indicates that the value of the hidden layer at instant *t*-1, $h_{{{\text{t}} - 1}}$, does not affect the value of the hidden layer at moment *t*, $h_{{\text{t}}}$. The update gate facilitates a better representation of the influence of data with a wider time range in the temporal series on the current moment. For the value $r_{{\text{t}}}$ of the reset gate at moment *t*, a larger value means that the candidate value $\tilde{h}_{{\text{t}}}$ at moment *t* is more influenced by the value $h_{{{\text{t}} - 1}}$ of the current concealed layer *t* a 1. If the value of $r_{{\text{t}}}$ is approximately zero, it means that the value of $h_{{{\text{t}} - 1}}$ in the covert layer at time *t*-1 does not contribute to the candidate value $\tilde{h}_{{\text{t}}}$ at time *t*. The reset gate helps to better reflect the influence of the shorter intervals in the temporal series on the current moment.

Given the input value $x_{{\text{t}}} \left( {t - 1,2, \ldots ,n} \right)$, the value of the hidden layers at instant *t* for the GRU neural network is [27]

11 $$z_{{\text{t}}} = g\left( {W_{z} \cdot \left[ {h_{{{\text{t}} - 1}} ,\;x_{{\text{t}}} } \right]} \right)$$

12 $$r_{{\text{t}}} = g\left( {W_{{\text{r}}} \cdot \left[ {h_{{{\text{t}} - 1}} } \right.,\;\left. {x_{{\text{t}}} } \right]} \right)$$

13 $$\tilde{h}_{{\text{t}}} = f\left( {W_{{\tilde{h}}} \cdot \left[ {r_{{\text{t}}} \bigcirc h_{{{\text{t}} - 1}} } \right.,\;\left. {x_{{\text{t}}} } \right]} \right)$$

14 $$h_{{\text{t}}} = \left( {1 - z_{{\text{t}}} } \right)\bigcirc h_{{{\text{t}} - 1}} + z_{{\text{t}}} \bigcirc \tilde{h}_{{\text{t}}}$$

where: "[]" means two vectors are connected; "○" is a calculation method between matrices, which means multiply by elements, when "○" acts on two vectors, the operation is

15 $$a\bigcirc {\text{b}} = \left[ {\begin{array}{*{20}c} {a_{1} } \\ {a_{2} } \\ {a_{3} } \\ \vdots \\ {a_{n} } \\ \end{array} } \right]\bigcirc \left[ {\begin{array}{*{20}c} {b_{1} } \\ {b_{2} } \\ {b_{3} } \\ \vdots \\ {b_{n} } \\ \end{array} } \right] = \left[ {\begin{array}{*{20}c} {a_{1} b_{1} } \\ {a_{2} b_{2} } \\ {a_{3} b_{3} } \\ \vdots \\ {a_{n} b_{n} } \\ \end{array} } \right]$$

From Eqs. (1) to (4), it can be seen that the weight matrices at time $t$ for which the GRU neural network needs to be trained are $W_{z}$, $W_{r}$ and $W_{{\tilde{h}}}$, which are combined by two weight matrices, respectively, i.e.,

16 $$W_{z} = W_{zx} + W_{{z\tilde{h}}}$$

17 $$W_{r} = W_{rx} + W_{{r\tilde{h}}}$$

18 $$W_{{\tilde{h}}} = W_{{\tilde{h}x}} + W_{{\tilde{h}\tilde{h}}}$$

where $W_{zx}$, $W_{rx}$ and $W_{{\tilde{h}x}}$ are the weight matrices of the input value to the update gate, the input value to the reset gate and the input value to the candidate value, respectively; $W_{zx}$, $W_{rx}$ and $W_{{\tilde{h}x}}$ are the weight matrices of the last candidate value to the update gate, a reset gate's last candidate value and a candidate value's latest candidate value, respectively.

The training method for GRU neural networks is based on back-propagation theory and consists of four main steps.

1. Forward computation of each neuron's output value.
2. Back-propagation of the error term for each neuron the back-propagation of the GRU neural network error term consists of two aspects: one is the back-propagation along time, i.e., the calculation of the error term for each moment from the current moment, and the other is the transfer of the error term to the previous layer.
3. Based on the error term, the corresponding weight gradient is calculated using the optimization algorithm.
4. Update the weights using the obtained gradients. In this paper, stochastic gradient descent (SGD) is used to calculate the weight gradients. The ordinary batch gradient descent (BGD) method computes all the samples in each iteration and then updates the gradient; the SGD algorithm computes a random set of samples and updates the gradient. Compared with the BGD algorithm, the SGD algorithm is able to avoid falling into local extremes during the computation process, but does not need to compute all samples in each iteration, which can balance computational efficiency and computational accuracy.

### Blockchained supply chain prediction

**Fig. 7:**
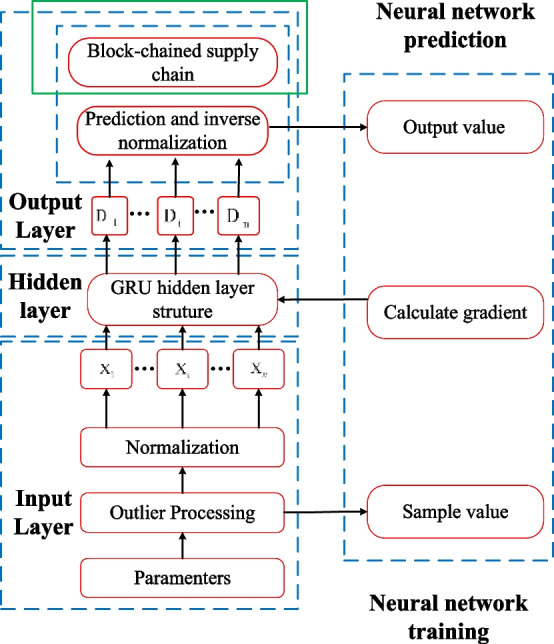
Prediction flow based on GRU neural network

Figure 7 shows the flow of blockchained supply chain prediction based on GRU neural network. A hidden layer, an output layer, and an input layer are present, as shown in the picture. The input layer performs outlier processing and normalization of the storage layer parameters and feeds the processed data into the hidden layer. The goal of normalization is to keep the input data's maximum and lowest values within the bounds of the functions for the hidden layer and the output layer. The normalization formula used in this paper is

19 $$\begin{array}{*{20}c} {\tilde{x}_{i} = \frac{{x_{i} - x_{{{\text{min}}}} }}{{x_{{{\text{max}}}} - x_{{{\text{min}}}} }}} & {i = 1,2, \ldots ,n} \\ \end{array}$$

where $x_{{{\text{min}}}}$ and $x_{{{\text{max}}}}$ are the minimum and maximum values of *x*_*i*_ respectively.

When training the neural network, the hidden layer receives the data and uses the constructed GRU neural network to calculate and pass the results to the output layer; the output layer receives the calculation results and performs denormalization to provide the output results; the output results are compared with the sample values and the weight coefficients of the hidden layer are iteratively updated until the end of training. When performing neural network prediction, the hidden layer receives the data and uses the trained GRU neural network to calculate and pass the calculation results to the output layer; the output layer receives the calculation results and performs the inverse normalization to provide the cross-wave velocity information.

### Multi-head attention mechanism

To further improve the performance, we add to GRU the concept of Multi-Head Attention (MHA). Their research discovered that it is advantageous to use multi-head attention for the queries, values, and keys by using an attention layer as a function, which maps a query and a set of key-value pairs to the output. The multi-head attention layer computes the hidden information by linearly projecting the context vectors into several subspaces, performing better than single-head attention. We generate the output using weighted values, which are determined by queries and the related keys.

The time-dimension calculation for attention weighting is given by

20 $$s_{{\text{t}}} = {\text{softmax}}\left( {o_{{\text{last }}} \times \left( {o_{{\text{all }}} \times W_{{\text{t}}} } \right)^{{\text{H}}} } \right),\quad o_{{\text{last }}} \in R^{{{\text{B}},1,{\text{Z}}}}$$

21 $$o_{{\text{t}}} = s_{{\text{t}}} \times o_{{\text{all }}} ,\;o_{{\text{all }}} \in R^{{{\text{B,}}\;{\text{T,}}\;{\text{Z}}}} ,\;s_{{\text{t}}} \in R^{{{\text{B}},\;1,\;{\text{T}}}}$$

where $s_{t}$ contributes the time-dimension's attention score, $o_{{\text{last }}}$ stands for the most recent output, and $o_{{\text{all }}}$ refers to the total output. $T$ stands for the number of time steps, $B$ for the batch size, and $Z$ for the feature dimension. The most recent time step is represented by parameter $1$. The output of the time-dimension attention layer is donated by $o_{t}$, while $H$ stands for the transpose operator, $W_{t}$ for the parameter matrix, and $W_{t}$ for the transpose operator.

Single-Head Attention calculation is shown in Eqs. 20 and 21. For attention, we simply employ two GRU output varieties. The fact that the output of all time comprises data from every GRU output makes it crucial. The last time step output was chosen since it has the most redundant data of all the time steps. In order to calculate the queries, keys, and values for multi-head time-dimension attention computing, we also select the following two forms of output:

22 $$K_{i} = W_{i,k} \times o_{{\text{all }}} + b_{i,k} ,\;K_{i} \in R^{{{\text{B,}}\;{\text{T}},\frac{{\text{Z}}}{n}}} ,\;W_{i,k} \in R^{{{\text{Z}},\frac{{\text{Z}}}{n}}} ,\;b_{i,k} \in R^{{\frac{{\text{Z}}}{n}}}$$

23 $${\text{V}}_{{\text{i}}} = {\text{W}}_{{{\text{i}},{\text{v}}}} \times {\text{o}}_{{\text{all }}} + {\text{b}}_{{{\text{i}},{\text{v}}}} ,\;{\text{V}}_{{\text{i}}} \in {\text{R}}^{{{\text{B}},{\text{T}},\frac{{\text{Z}}}{{\text{n}}}}} ,\;{\text{W}}_{{{\text{i}},{\text{v}}}} \in {\text{R}}^{{{\text{Z}},\frac{{\text{Z}}}{{\text{n}}}}} ,\;{\text{b}}_{{{\text{i}},{\text{v}}}} \in {\text{R}}^{{\frac{{\text{Z}}}{{\text{n}}}}}$$

24 $$Q_{i} = W_{i,q} \times o_{{\text{last }}} + b_{i,q} ,\;Q_{i} \in R^{{{\text{B}},1\frac{{\text{Z}}}{n}}} ,\;W_{i,q} \in R^{{{\text{Z}},\frac{{\text{Z}}}{n}}} ,\;b_{i,q} \in R^{{\frac{{\text{Z}}}{n}}}$$

where $K,V,Q$ represent the value, key, and query, respectively. $n$ is the number of attention heads and $b$ means bias.

**Fig. 8:**
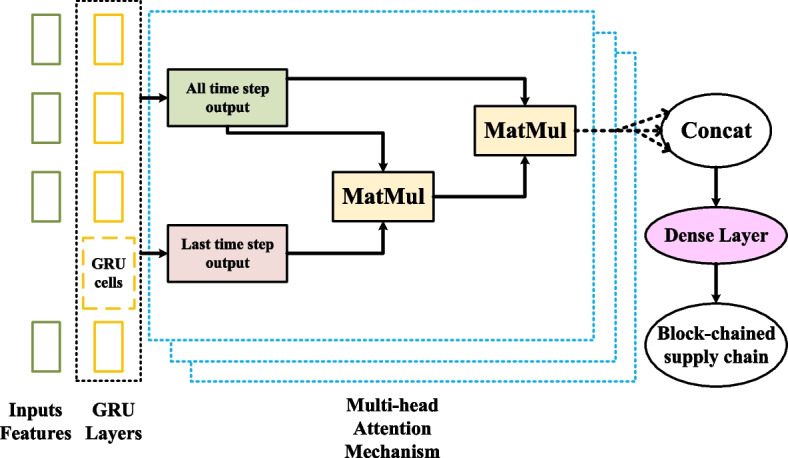
Structure of Multi-Head Attention GRU model

The following formulas are used to calculate the context vectors and C for Multi-Head Attention scores.

25 $$s_{i} = {\text{softmax}}\left( {Q_{i} \times K_{i}^{{\text{H}}} } \right),\;s_{i} \in R^{{\text{B,1,T}}}$$

26 $${\text{context}}_{i} = s_{i} \times V_{i} ,\;{\text{context}}_{i} \in R^{{{\text{B}},1,\frac{{\text{Z}}}{n}}}$$

27 $$C = {\text{ Concat }}\left( {\left[ {{\text{context}}_{1} , \ldots ,{\text{context}}_{n} } \right]} \right),\;C \in R^{{\text{B,1,Z}}}$$

where ${\text{context}}_{i}$ denotes the reduced-dimension ${\text{context}}_{i}$ vectors from each subspace and $s_{i}$ denotes the multi-head time-dimension attention score. Figure 8 illustrates the general organization of multi-head time-dimension attention. The context vector is then inserted into the complete connection layer. The softmax layer receives the output and makes the final prediction.

## Results and discussion

Incorporating machine learning into supply chain management can aid in automating a lot of tedious processes and free up businesses to concentrate on more strategic and significant commercial endeavors. Supply chain managers can locate the best suppliers and optimize inventory using clever machine learning tools to keep their business running smoothly. As a result of machine learning's many benefits and the opportunity to fully utilize the enormous volumes of data gathered by warehousing, transportation systems, and industrial logistics, an increasing number of enterprises are now exhibiting interest in its applications. Additionally, it may assist businesses in building a complete supply chain model that is powered by machine intelligence in order to reduce risks, increase insights, and improve performance—all of which are essential for developing a supply chain model that is globally competitive.

In this section, we will demonstrate the performance of different machine learning tools in term of demand prediction of IoT tracked refrigerators. It’s worth noting that the same method can be used to forecast product quality, product lifecycle, etc. as well.

The model is trained using the training set, and the test set's prediction value is acquired once the model has been trained. The fitted and projected results are compared using the Root-Mean-Square Error (RMSE), and Eq. (20) provides its definition. The accuracy of the model increases with decreasing RMSE.

28 $${\text{RMSE}} = \sqrt {\frac{{\mathop \sum \nolimits_{i = 1}^{n} \left( {\widehat{{y_{i} }} - y_{i} } \right)^{2} }}{n}}$$

where $y_{i}$ is the actual data, $\widehat{{y_{i} }}$ is the model's projected value, and $n$ is the total sample size (Table 1).

**Table 1:** Parameter setup for different machine learning methods

Parameter	GRU	RNN
Network structure	Data normalization multi-head attention—GRU (three head)	Data normalization three layers RNN
Learning rate(lr)	0.01	0.01
lr scheduler	MultiStepLR	MultiStepLR
Optimizer	SGD	SGD
Loss function	RMSE	RMSE
Batch size	56	56
Epoch	80	80
Randomseed	0	0

**Fig. 9:**
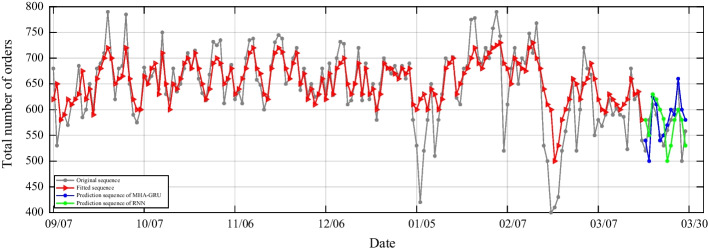
RNN versus MHA-GRU: prediction results of demand

We use a sliding window to produce the drill set and the testing set while applying RNN and MHA-GRU to forecast the order number. The data from the second day are utilized as a designation after the data from the first day were used as input. Both the RNN and MHA-GRU utilized in this study have three layers. Prior doing anything else, the inputs must be normalized. The RNN and MHA-GRU models should then be trained using the practice set. Finally, inversely normalize the test set's outputs to get the projected order number. In Fig. 9, the prediction results are shown. The RNN and MHA-GRU prediction accuracy are described in Fig. 9 in the meanwhile.

Figure 9 reveals that MHA-GRU model has a more stable curve when the fitting accuracy of RNN and MHA-GRU are compared. The red curve in Fig. 9 is the fitted curve for stability test. The fitted curve can roughly keep consistent with the overall trend of the original curve, while providing guarantee for the subsequent curve prediction of MHA-GRU and RNN. The blue and green curves in Fig. 9 represent the effect of inverse normalization after prediction of MHA-GRU and RNN, respectively. It can be clearly observed from that the blue curve fits the trend very closely to the direction of the original data, while on the contrary, the green curve tends to express above or below the original curve. RNN cannot handle the long-term dependencies due to vanishing/exploding gradient problem, and then MHA-GRU is introduced to overcome this shortcoming.

**Fig. 10:**
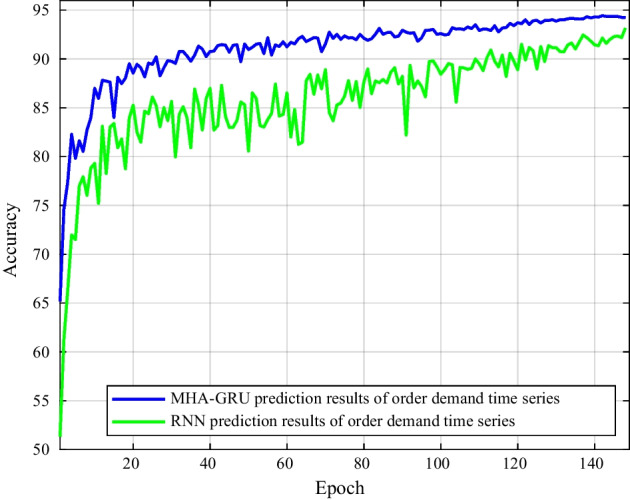
Prediction accuracy of RNN and MHA-GRU

Figure 10 illustrates the accuracy curves of MHA-GRU and RNN predictions. Apparently MHA-GRU model provides a better accuracy of prediction for the series, as it has superior fitting and prediction accuracy. Predicting the short-term tendency of client volume can help significantly supply chain managers make good commercial choices, increase management effectiveness, and become more responsive to market changes.

## Conclusion

Using cutting-edge IoT tracking technologies, this work conducts extensive research on supply chain management. In particular, we leverage machine learning over blockchained big data. Modern supply chain management based on machine learning is capable of achieving self-optimization and continual improvement to assure sustained growth. Blockchain technology can compensate for poor safety and privacy, lack of trust in online transactions, and inadequate protection of property rights. Consequently, manufacturers are able to make accurate forecasts of customer demand, formulate flawless production plans, and coordinate all parties and links in the supply chain to achieve an integrated arrangement and efficient management, thereby maximizing profits.

## Data Availability

Data is confidential and not public.

## Abbreviations

IoT: Internet of things
ML: Machine learning
LSTM: Long short-term memory network
RNN: Recurrent neural network
SGD: Stochastic gradient descent
BGD: Batch gradient descent
AIC: Akaike information criterion
